# PD26 Seeking Sustainability For The Brazilian Public Healthcare System: Cost-Utility Analysis Of Pembrolizumab For Advanced Non-Small Cell Lung Cancer

**DOI:** 10.1017/S0266462324002903

**Published:** 2025-01-07

**Authors:** Ludmila Gargano, Cecilia Farinasso, Haliton Alves De Oliveira Junior, Rosa Lucchetta

## Abstract

**Introduction:**

Pembrolizumab is used as a monotherapy or in addition to chemotherapy for patients with non-small cell lung cancer (NSCLC) as an alternative to platinum-based chemotherapy. The objective was to conduct a cost-utility analysis for subgroups of patients with NSCLC using the Brazilian Unified Health System (SUS) threshold of BRL120,000 (USD24,458) per quality-adjusted life-year (QALY).

**Methods:**

We built a partitioned survival model with three health states based on overall survival (OS) and progression-free survival (PFS) curves for three scenarios according to programed death ligand-1 (PD-L1) expression as follows: (i) pembrolizumab monotherapy for at least 50 percent PD-L1; (ii) pembrolizumab monotherapy for at least one percent PD-L1; and (iii) pembrolizumab plus chemotherapy for one to 49 percent PD-L1. The outcome of interest was QALYs, so utility values were derived from pre- and post-progression states according to the treatment received. Survival curves were extrapolated for 20 years using different distributions. The best fitted distribution was selected by visual inspection, clinical plausibility, and Akaike and Bayesian information criterion tests. Direct costs were also considered.

**Results:**

Pembrolizumab provided incremental gains of 1.23, 0.35, and 1.10 QALYs when compared with platinum-based chemotherapy for scenarios one, two, and three, respectively. The subgroup with the best incremental cost-utility ratio (ICUR) was pembrolizumab monotherapy for at least 50 percent PD-1 (BRL201.366 [USD41,041] per QALY gained), followed by pembrolizumab with chemotherapy for one to 49 percent PD-L1 (BRL267.216 [USD54,463] per QALY gained). When assessed for all PD-L1 positive patients, the ICUR reached BRL571.425 (USD116,465), which was 4.5 times the cost-effectiveness threshold. Sensitivity analysis showed that pembrolizumab must cost a maximum of BRL4.761 (USD970) per vial to be cost effective for the SUS, which is a 40.7 percent reduction in the base case price.

**Conclusions:**

Evidence shows that pembrolizumab alone or in combination with chemotherapy is more effective than platinum-based chemotherapy for treating patients with NSCLC, especially those with a high expression of PD-L1. However, price reduction is essential for pembrolizumab to be cost effective for treating these patients in the SUS.

